# When You Become a Superman: Subliminal Exposure to Death-Related Stimuli Enhances Men’s Physical Force

**DOI:** 10.3389/fpsyg.2018.00221

**Published:** 2018-02-28

**Authors:** Naoaki Kawakami, Emi Miura, Masayoshi Nagai

**Affiliations:** ^1^Faculty of Human Sciences, Shimane University, Matsue, Japan; ^2^Graduate School of Education, Shimane University, Matsue, Japan; ^3^College of Comprehensive Psychology, Ritsumeikan University, Ibaraki, Japan

**Keywords:** terror management theory, unconscious processes, subliminal priming, physical force, muscularity

## Abstract

Research based on terror management theory (TMT) has consistently found that reminders to individuals about their mortality engender responses aimed at shoring up faith in their cultural belief system. Previous studies have focused on the critical role that the accessibility of death-related thought plays in these effects. Moreover, it has been shown that these effects occur even when death-related stimuli are presented without awareness, suggesting the unconscious effects of mortality salience. Because one pervasive cultural ideal for men is to be strong, we hypothesized that priming death-related stimuli would lead to increasing physical force for men, but not for women. Building on self-escape mechanisms from TMT, we propose that the mechanism that turns priming of death-related stimuli into physical exertion relies on the co-activation of the self with death-related concepts. To test this hypothesis, we subjected 123 participants to a priming task that enabled us to combine the subliminal priming of death-related words with briefly presented self-related words. Accordingly, three different conditions were created: a (control) condition in which only self-related stimuli were presented, a (priming) condition in which death-related words were subliminally primed but not directly paired with self-related stimuli, and a (priming-plus-self) condition in which death-related words were subliminally primed and immediately linked to self-related stimuli. We recorded handgrip force before and after the manipulations. Results showed that male participants in the priming-plus-self condition had a higher peak force output than the priming and control conditions, while this effect was absent among female participants. These results support the hypothesis that unconscious mortality salience, which is accompanied with self-related stimuli, increases physical force for men but not for women. The gender difference may reflect the cultural belief system, in which individuals are taught that men should be strong. Thus, the unconscious mortality salience produced by exposure to the death-related stimuli motivates need to conform to this internalized cultural standard.

## Introduction

In February 2012, a 15-year-old Michigan boy and his grandfather were fixing an old car. While the boy was inside the car, it shifted and collapsed, pinning his grandfather under 900 kg of steel. The boy was extremely scared and did not know what to do, but he took action. Surprisingly, the boy was able to lift the front end of the car high enough so that his grandfather could crawl out (WDIV Detroit, 2012). In this way, people sometimes can muster extraordinary strength, more than they think they have. These phenomena are considered to be caused by increased arousal in the face of a threat to life (Perkins et al., 2001).

In the field of sport psychology, it is well known that athletes often use performance enhancement techniques of some kind, or “psyching up,” to increase arousal in an attempt to facilitate performance (Perkins et al., 2001), particularly when there is a need for explosive force production (Tod et al., 2003). The term “psyching-up” can be defined as the use of cognitive and/or somatic techniques that induce strong arousal, designed to enhance performance, before or during competition (Tod et al., 2003). Such studies have assessed the effect of imagery, attentional focus, self-efficacy statements, and preparatory arousal (i.e., raising arousal by asking participants to get themselves emotionally “pumped up” or “charged up”; Shelton and Mahoney, 1978; Weinberg et al., 1980; Caudill et al., 1983). These and other studies generally indicate that preparatory arousal produces the greatest increases in maximal strength performance (Gould et al., 1980; Seabourne et al., 1985; Tynes and McFatter, 1987; Whelan et al., 1990).

However, a recent study suggests that the use of cognitive and/or somatic techniques, such as psyching up, to induce strong conscious arousal is not necessarily required to enhance physical force (Aarts et al., 2008). In this study, participants had to squeeze a handgrip as hard as they could in response to a start sign. Prior to this task, words pertaining to the action concept of physical exertion (e.g., exert, vigorous) were subliminally presented or not presented, together with consciously visible positive words that signal rewards (e.g., good, nice) or not^1^ Results demonstrated that participants who were subliminally exposed to exertion with a positive reward signal showed more forceful squeezing although consciously reported motivation and arousal did not show any relation to the subliminal priming manipulation. This study indicates that unconscious and purely cognitive triggers, independently of conscious arousal, can increase physical force. Sport psychologists have searched for new mechanisms to unlock the full potential of physical force; hence, identifying such a mechanism in an experimental situation would be useful in understanding physical exertion and athletic performance outside of an experimental context. We approached this issue from a different perspective, namely, terror management theory.

### Terror Management Theory and Research

Terror management theory (TMT; Greenberg et al., 1986; Solomon et al., 1991) posits that the juxtaposition of the awareness that death is inevitable with the desire for survival is the most fundamental threat to the human self. According to the dual-process model of TMT (Pyszczynski et al., 1999), once people experience death-related thoughts inside of current focal attention, they try to remove death-related thought from consciousness and/or push death into the distant future (i.e., a proximal defense). However, after this defense succeeds in removing death concerns from focal attention, or when death concerns are activated outside of conscious awareness (i.e., subliminally), people engage distal defenses that serve to assuage unconscious mortality concerns through symbolic protection from death not only on a social level (i.e., defending cultural worldviews) but also on a personal level (i.e., enhancing their self-esteem). In the last 25 years, over 200 studies have been reported supporting two hypotheses derived from TMT on the social and personal levels (see Burke et al., 2010, for a review).

The first hypothesis is that reminders to individuals about their mortality motivate them to engage in cognitive and behavioral efforts aimed at validating their cultural worldviews, allowing individuals to understand and give meaning to the world they live in and thereby gain a sense of value along with a promise of symbolic immortality (see Greenberg et al., 1997, for a review). For instance, reminders to participants of their mortality increases such responses as positive evaluations of those who validate the participants’ belief system and negative evaluations of those who threaten it (e.g., Rosenblatt et al., 1989), perceived consensus for participants’ beliefs (Pyszczynski et al., 1996), reluctance to violate cultural norms (Greenberg et al., 1995), stereotypical thinking and preferences (Schimel et al., 1999), and aggression against those who violate their cultural worldview (McGregor et al., 1998). These responses are posited to attenuate existential concerns engendered by thoughts of death by weaving the individual more securely into a meaningful cultural fabric.

The second hypothesis is that reminders to individuals about their mortality engender cognitive and behavioral efforts aimed at increasing self-esteem (Solomon et al., 1991). From the perspective of TMT, self-esteem is a culturally derived construction that is dependent on sources of social validation, and it functions to buffer anxiety concerning one’s mortality by living up to the standards of value endorsed by the individual’s society and culture (Pyszczynski et al., 2004). Indeed, a large body of research has shown the cognitive ramifications associated with increased self-esteem strivings in response to reminders of mortality (Routledge et al., 2004; Goldenberg and Arndt, 2008). For instance, mortality salience leads to overestimation of future financial worth (Kasser and Sheldon, 2000) and increased favorability toward charities (Jonas et al., 2002). Moreover, mortality salience increases intentions to exercise (Arndt et al., 2003), identification with physical bodies (Goldenberg et al., 2000, Study 1), and interest in sex (Goldenberg et al., 2000, Study 2), but only among participants whose self-esteem is partially derived from such domains. On the other hand, when one’s group identifications (e.g., gender identity) are likely to reflect negatively on one’s self-esteem, mortality salience leads participants to move away from their in-group, even at the expense of belongingness or in-group favoritism (Arndt et al., 2002b). Taken together, these and other studies (see Pyszczynski et al., 2004, for a review) support the hypothesis that because self-esteem helps defend people from awareness of mortality, people engage in cognitive and behavioral efforts aimed at increasing or at least maintaining self-esteem when they experience death-related thoughts. The present study focuses on this self-esteem striving mechanism and examines its manifestations on physical strength.

Indeed, some studies have demonstrated that mortality salience manipulations affect behavioral domains that function to boost self-esteem. A series of studies by Taubman Ben-Ari et al. (1999) showed that mortality salience increased risky driving behavior on a driving simulator among participants who valued their driving ability as a source of self-esteem. Moreover, mortality salience increased interest in tanning products and services among female participants who were primed to associate tanned skin with an attractive appearance (Routledge et al., 2004). These findings suggest the possibility that mortality salience could influence a more fundamental behavioral domain, namely physical strength, but only if physical strength provides individuals with a source of self-esteem.

### The Muscularity Ideal for Men

One pervasive cultural ideal for men is to be strong. In contrast to women, who experience pressure to be slender, men experience pressure to maintain a muscular body as a symbol of physical strength. Research assessing men’s concerns with their muscularity found that 84% of college men and 44% of older men indicated that their current bodies were less muscular than the bodies they would like to possess (Lynch and Zellner, 1999). The ideal example is Superman. Many children yearn to be Superman, who battles for peace through incredible force (he can lift an object of 800,000 tons!). In fact, men represented as prestigious in popular magazines are often muscular (e.g., Frederick et al., 2005). Moreover, past research has found that men in several cultures desired bodies that were more muscular than their current bodies and generally believed that women prefer men who are more muscular than less muscular men (Lipinski and Pope, 2002; Olivardia et al., 2004; Campbell et al., 2005; Yang et al., 2005; Crossley et al., 2012; Uragami et al., 2013). Additionally, from an evolutionary perspective, being physically strong with a muscular body type is desirable for several reasons (Frederick and Haselton, 2007; Frederick et al., 2007). First, physical strength aids men in male–male intrasexual competition, allowing men with muscular bodies to achieve higher status among males. Second, possessing a muscular body as a symbol of physical strength may be an important component of male attractiveness to women across a variety of cultures. Although high physical strength is not synonymous to a socially attractive lean-muscled body, physical strength can be an indirect indicator of muscular body. In this way, theory, research, and common observation suggest that high physical strength, as a potential source of the perception of a muscular body, is a particularly important domain that enables men to maintain the meaning and value of the self. Given that TMT posits that reminders of death should lead to an increased need for self-esteem and thus increased efforts to live up to the standards of value from which one’s self-esteem derives, mortality salience should fuel men’s motivation to be strong. In fact, previous research has shown that the thin ideal, a particularly salient standard for most women, could guide women’s striving to attain a thin physique following a reminder of mortality (Goldenberg et al., 2005). In the present study, we investigated the possibility that mortality salience leads men to exercise their potential physical strength more than they think they are able.

### TMT and Self-Awareness

On the other hand, recent research by Wisman et al. (2015) showed that when thoughts about death are accessible outside of conscious awareness, people low in self-esteem are motivated to increase behaviors (i.e., writing about others than themselves and consuming alcohol) that are associated with escaping the self rather than increase behaviors that are associated with bolstering self-esteem. Because people who lack self-esteem in a certain self-aspect cannot identify more with that construct, they are instead prone to shutting down the self by avoiding self-awareness when mortality is salient (Wisman, 2006). Namely, pushing the self away from death can facilitate the suppression of fear of death, defending them from mortality concerns. For example, Goldenberg et al. (2000) found that mortality salience decreased monitoring of one’s appearance among participants low in body-esteem whereas participants high in body-esteem responded to mortality salience by identifying more highly with their bodies. Other studies also suggest that self-awareness is particularly aversive in the context of mortality salience and people tend to avoid self-awareness, to buffer against death thoughts (Arndt et al., 1998). In light of the fact that the majority of college men have low muscularity esteem, as described above (Lynch and Zellner, 1999), a mortality salience manipulation may motivate men to escape self-awareness especially in the muscularity domain and may not lead them to exercise their potential physical strength to bolster their self-esteem. Therefore, we propose and test the effect of co-activation of the self and death in order to turn a mortality salience manipulation into men’s physical exertion. As the objective self-awareness (OSA) theory (Duval and Wicklund, 1972) suggests, focusing attention on the self can be one of the important motives of behavior control (Carver and Scheier, 1981, 1998). For instance, high self-focus individuals are more likely to be honest, helpful, and industrious in their behavior (Wicklund and Frey, 1980). Appling this perspective to TMT, we predict the following mechanism: based on TMT, mortality salience would lead to increased need for self-esteem. At the same time, however, it would motivate people who lack self-esteem in a certain self-aspect to escape the self, resulting in them not increasing behaviors directed at bolstering self-esteem in that self-aspect domain. Nevertheless, when the self is co-activated and directly associated with death, people can no longer escape from self-awareness and the fear of self-demise, which, in turn, should fuel behaviors that are directed at bolstering their self-esteem.

### The Present Study

The present study was designed to test whether mortality salience would lead to an increase in physical force for men, but not for women. According to the influence of mortality salience on self-awareness, however, men’s physical force would increase only when reminders of death are associated with self-related stimuli acting as a mediator.

As the mortality salience induction, we used a subliminal priming technique. Based on TMT, distal defenses serving to engender cognitive and behavioral efforts aimed at increasing self-esteem are activated when thoughts of mortality are not consciously accessible (Arndt et al., 1997a, 2002b; Burke et al., 2010). For example, Greenberg et al. (1994) demonstrated that explicitly reminding participants of their death (either with a writing task or with supraliminal word presentations) increased worldview accessibility only after a delay, at a time point when previous research has shown that death-thought accessibility to be high but outside of focal attention (Arndt et al., 1997b). More directly, Arndt et al. (1997a) demonstrated that participants who were presented with death-related primes outside of conscious awareness via subliminal priming techniques showed more favorable evaluations of people who praised participants’ cultural worldviews and more unfavorable evaluations of those who challenged it. This and other studies have demonstrated that the same mortality salience effects have been repeatedly obtained with subliminal and supraliminal priming manipulations alike.

With regard to the use of a subliminal priming technique in the present study, there are three main advantages. First, subliminal priming techniques help us to control what exactly is primed, and to ensure that obtained effects are not due to experimental demand or some other intentional and strategic processing by the participant (Bargh, 1994). On the other hand, in traditional death reminders (e.g., supraliminal priming of death-related words and open-ended questions about death), participants can know what is primed. This could cause the participant to activate various factors other than the factor that the researcher focuses on. In our experiment, specifically, it is possible that if death reminders were to be perceived consciously, they might act as negative primes on the self rather than as a mortality salience manipulation. Second, subliminal priming hardly produces conscious arousal compared to supraliminal priming (Aarts et al., 2008). Although no evidence of mediation by consciously experienced affect or physiological arousal has been found in TMT studies (Greenberg et al., 1997; Pyszczynski et al., 1999; Arndt et al., 2001), ensuring independence of conscious arousal and mortality salience is important for us because one of our purposes is to show the enhancement of physical force without the benefit of strong conscious arousal, such as occurs in the psyching up technique. Finally, subliminal priming techniques help us directly to show the unconscious processes underlying the effect of mortality salience. Given the ubiquity with which people may confront situations that subtly provoke death-related thought (e.g., proximity to a funeral parlor), it is worthwhile to demonstrate the automaticity of physical strength enhancement as a symbolic defense against mortality concerns in the experimental context.

In sum, we conducted a priming experiment that combined a mortality salience induction using subliminal priming of words representing death with briefly presented self-related words. To investigate the effect of the association between self and death more specifically, all participants were exposed to self-related words. Accordingly, three priming conditions were created: a (control) condition in which only self-related stimuli were presented, a (mortality salience; MS) condition in which death-related stimuli were subliminally primed but not directly paired with self-related stimuli, and a (MS-plus-self) condition in which death-related stimuli were subliminally primed and immediately linked to self-related stimuli. Therefore, we hypothesized a three-way interaction such that mortality salience would lead men but not women to increase physical force from pre-manipulation to post-manipulation (as measured by a hand dynamometer) in the condition in which death is directly associated with the self (MS-plus-self condition), but not in the conditions in which death is not directly associated with the self (MS condition) and in which death is no longer primed (control condition).

## Materials and Methods

### Participants and Design

One hundred twenty-three undergraduate students (62 females; mean age = 20.82 years, range = 18–24) participated voluntarily in the experiment. The experiment consisted of a 3 (condition: control vs. MS vs. MS-plus-self) × 2 (gender: male vs. female) × 2 (measurement: pre-manipulation vs. post-manipulation) mixed design. The latter factor was a within-participants factor. The study was approved by the local Ethics Committee of the University of Tsukuba. All subjects gave written informed consent in accordance with the Declaration of Helsinki.

### Apparatus

This experiment was conducted on a desktop PC and a 17-inch 100-Hz CRT screen. The viewing distance was approximately 70 cm. A Toei hand dynamometer (Model # T-2177) with a range of 0–100 kg was employed to measure handgrip force.

### Materials

As primes, on the basis of previous studies, five death-related words (i.e., death, grave, dying, killing, and cadaver), and five nonsense syllables were selected. As associative stimuli, we used five self-related words (i.e., I, myself, mine, participant’s first name, and participant’s last name), and five neutral words that were clearly unrelated to the concept of self.

### Procedure

The experiment was conducted individually, and described as one examining the relation between physical force and visual information processing. The experimenters were blind to the hypotheses and conditions. First, participants performed a handgrip-force task as a pre-manipulation measure. They were asked to squeeze the handgrip dynamometer with their dominant hand as hard as they could when the word “squeeze” appeared on the screen, and to stop squeezing when the word was erased (after 3500 ms). This task was repeated twice. Participants did not receive performance feedback. Then, they were asked to indicate how hard they tried to squeeze into the handgrip (1 = not at all, 5 = very much), and asked to answer the Affect Grid that was designed simultaneously to assess subjectively felt valence and arousal (Russell et al., 1989). The Affect Grid is a visual 9 × 9 two-dimensional grid, with unpleasant/pleasant feelings forming the horizontal ranging from 1 to 9, and sleepiness/arousal forming the vertical dimension ranging from 1 to 9, with end points and neutral points marked with emotion words to facilitate reporting. Each position on the grid thus corresponds to a particular valence and arousal score.

After the pre-manipulation measure, the priming experiment started with a task designed to combine subliminal priming of the concept of death with self-related stimuli on the basis of the technique by Custers and Aarts (2005). In this task, participants had to detect dots that were presented either above or below (self-related and neutral) words briefly presented on the screen. This feature of the task ensured that participants attended to the post masked subliminal primes. Participants practiced the task with unrelated stimuli words, and then worked on 50 experimental trials.

**FIGURE 1:**
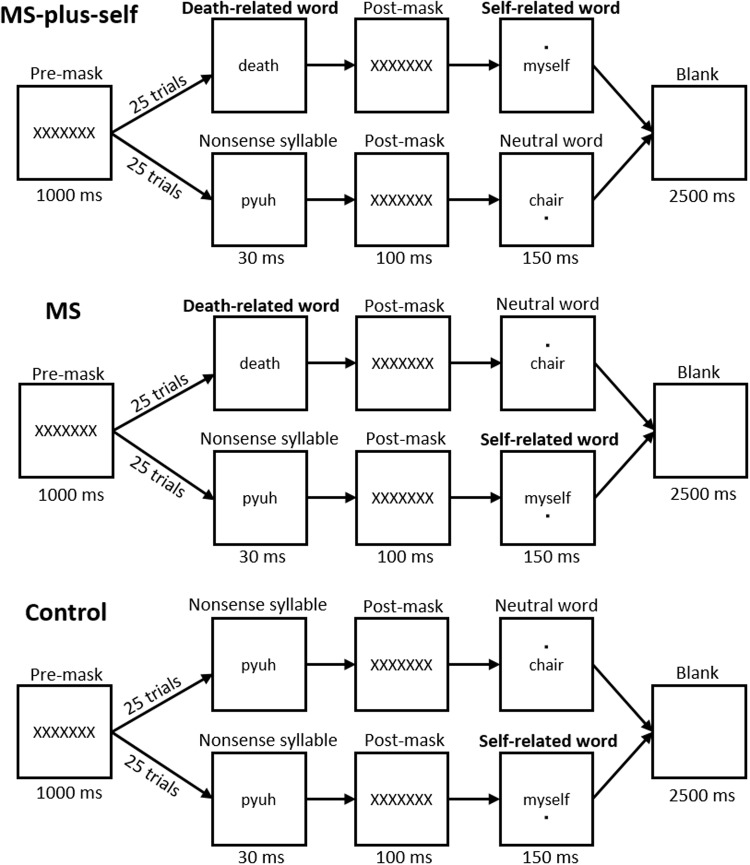
Sequence of events for each condition.

In these 50 trials (see **Figure 1**), the five self-related and five neutral words were all presented five times. In the MS-plus-self condition, the five death-related words were each presented subliminally on five trials in direct combination with the five different self-related words (25 trials), and five nonsense syllables were presented in combination with the five different neutral words (25 trials). In the MS condition, the death-related words were paired with neutral words (25 trials), and the nonsense syllables were paired with self-related words (25 trials). In a control condition, five nonsense syllables were paired with five self-related (25 trials) and five neutral words (25 trials). In each condition, namely, participants were exposed to neutral and self-related words. The only difference was that the mortality salience was paired directly with the self (MS-plus-self condition), primed but not directly linked to the self (MS condition), or not primed at all (control condition). The order of the 50 trials was randomized.

Each trial consisted of the following events (see **Figure 1**): A pre-mask (i.e., XXXXXXX) presented for 1000 ms, signaling the beginning of the trial, immediately followed by the subliminal prime word—either a death-related word or nonsense syllable—displayed for 30 ms^2^. Thereafter, a post-mask (i.e., XXXXXXX) appeared again for 100 ms, followed by either a neutral or a self-related word that was presented for 150 ms. Occasionally, a dot was presented for 30 ms (not post-masked, hence consciously visible), either above or beneath the neutral or self-related word. Participants indicated whether they had seen a dot or not, and 2500 ms later, a new trial started.

After the priming task, participants were asked to perform a handgrip-force task twice in the same manner as in the pre-manipulation measure. Finally, they were asked again to indicate their physical effort toward the handgrip task, and to answer the Affect Grid.

## Results

### Manipulation Check

To check subliminality of the primes, we asked participants to answer the following questions: “Did you see something?” “If you had seen something, what did you see?” “Did you concentrate on the dot detection task?” and “Did you feel any suspicion during the dot detection task?” Most of the participants did not indicate awareness of the primes, or suspicion of any sort. Although five participants reported that they had seen at least one of the death-related words, we did not exclude their data because the following results were not changed^3^.

Following this, we analyzed the psychological measures, that is, arousal and feeling. The mean scores are shown in **Table 1**. We conducted 3 (condition: control vs. MS vs. MS-plus-self) × 2 (gender: male vs. female) × 2 (measurement: pre-manipulation vs. post-manipulation) ANOVAs on both the arousal and feeling scores from the Affect Grid. Crucially, no significant three-way interactions emerged, *F*(2,117) = 0.40, *p* = 0.67, ${\text{η}}_{\text{p}}^{2}$ = 0.01, and *F*(2,117) = 0.84, *p* = 0.84, ${\text{η}}_{\text{p}}^{2}$ = 0.00, res*p*ectively. These findings indicate that subliminal exposure to death-related stimuli did not influence participants’ conscious arousal level and feeling.

**Table 1:** Mean and standard deviation of arousal, feeling, and physical effort as a function of experimental condition, gender, and measurement.

	Condition					
	MS + Self		MS		Control	
Measure	*M*	*SD*	*M*	*SD*	*M*	*SD*
Arousal (range: 1–9)						
Male						
Pre	5.76	1.18	5.95	1.75	5.76	1.34
Post	5.86	1.31	5.63	1.74	5.62	1.60
Female						
Pre	5.65	1.76	5.90	1.14	6.00	1.82
Post	5.70	1.89	6.05	1.16	5.76	1.70
Feeling (range: 1–9)						
Male						
Pre	5.24	1.00	5.37	1.16	5.24	1.00
Post	5.29	1.01	5.26	1.15	5.19	0.81
Female						
Pre	5.05	1.00	5.19	1.17	5.24	1.41
Post	5.15	1.09	5.24	1.09	5.14	1.53
Physical effort (range: 1–5)						
Male						
Pre	4.71	0.46	4.80	0.41	4.71	0.46
Post	4.76	0.44	4.80	0.41	4.76	0.44
Female						
Pre	4.70	0.47	4.57	0.51	4.57	0.75
Post	4.60	0.50	4.71	0.46	4.52	0.81

Moreover, a 3 × 2 × 2 ANOVA on physical effort showed that there was no significant difference (see **Table 1**), *F*(2,117) = 1.12, *p* = 0.33, ${\text{η}}_{\text{p}}^{2}$ = 0.02, indicating that the subliminal priming of the death-related stimuli did not lead to an increase in conscious awareness of exerting physical effort.

### Main Results

To assess the effect of subliminal exposure to death-related stimuli, we calculated the individual mean of two pulls within each handgrip-force measure. Härkönen et al. (1993) found no statistical difference between one pull, and two pulls performed on a handgrip dynamometer. There were no outliers whose handgrip force differed three standard deviations or more from the mean of the participants in each condition.

**FIGURE 2:**
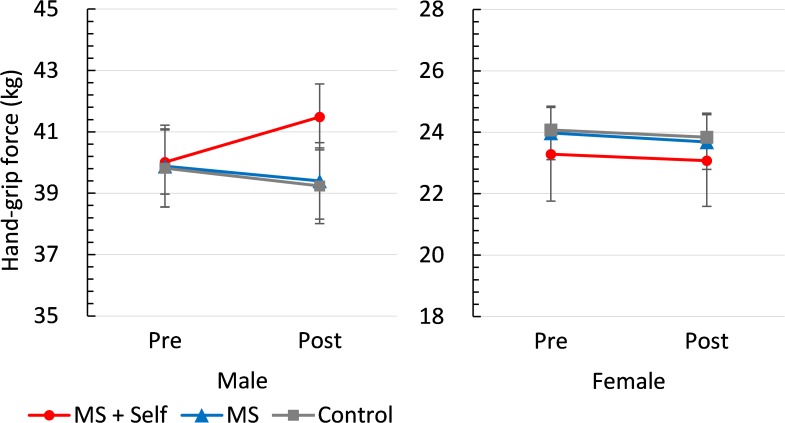
Mean handgrip force as a function of condition, gender, and measurement. The bars represent standard errors of the mean.

**Figure 2** summarizes the main results of the experiment. We conducted a 3 (condition: control vs. MS vs. MS-plus-self) × 2 (gender: male vs. female) × 2 (measurement: pre vs. post) ANOVA on the handgrip-force data. This analysis yielded main effects of gender and measurement, showing a higher force in male participants than in female participants, *F*(1,117) = 313.95, *p* = 0.000, ${\text{η}}_{\text{p}}^{2}$ = 0.73. Moreover, there was a significant condition × measurement interaction, *F*(2,117) = 4.60, *p* = 0.012, ${\text{η}}_{\text{p}}^{2}$ = 0.07, indicating that the participants in the MS-plus-self conditions showed a higher handgrip force at post-measurement than at pre-measurement. However, these effects were qualified by a significant three-way interaction, *F*(2,117) = 4.12, *p* = 0.019, ${\text{η}}_{\text{p}}^{2}$ = 0.07.

To interpret this three-way interaction, we conducted 3 (condition: control vs. MS vs. MS-plus-self) × 2 (measurement: pre vs. post) mixed design ANOVAs separately for the male participants and the female participants. Among male participants, we found the predicted condition × measurement interaction, *F*(2,58) = 6.11, *p* = 0.004, ${\text{η}}_{\text{p}}^{2}$ = 0.17, and no significant main effects. Testing the simple effects revealed that in the MS-plus-self condition, the participants showed a significant increment of their handgrip force, *F*(1,58) = 12.11, *p* = 0.002, ${\text{η}}_{\text{p}}^{2}$ = 0.38, whereas no such effect was observed for the control and MS conditions, *F*(1,58) = 0.67, *p* = 0.42, ${\text{η}}_{\text{p}}^{2}$ = 0.04, and *F*(1,58) = 2.00, *p* = 0.17, ${\text{η}}_{\text{p}}^{2}$ = 0.09, respectively. Among female participants, on the contrary, there were no significant main effects and interaction, *F*s < 2.00, *p*s > 0.10, ${\text{η}}_{\text{p}}^{2}$s < 0.03. Moreover, we conducted a 3 (condition: control vs. MS vs. MS-plus-self) × 2 (gender: male vs. female) between-participants design ANOVA separately for the pre- and post-measurements, showing no interaction, *F*(2,117) = 0.11, *p* = 0.90, ${\text{η}}_{\text{p}}^{2}$ = 0.00, and *F*(2,117) = 1.06, *p* = 0.35, ${\text{η}}_{\text{p}}^{2}$ = 0.02, respectively.

## Discussion

We demonstrated that unconscious mortality salience can enhance physical force among men but not among women when subliminal primes are accompanied by a self-related stimulus. However, it was found that unconscious mortality salience that was not linked directly to the self, did not show enhancement of physical force among both men and women. Rather, they showed decreased handgrip force. In light of the same tendency in the control condition, it is supposed that this was due to muscle fatigue. Since our experimental design regarding measurement was within factor, the participants had to pull the handgrip dynamometer 4 times in total in a short span, resulting in exhaustion of the participants. This result suggests that a standard mortality salience manipulation did not show the corresponding effects of TMT. On the other hand, death-related stimuli in the MS and the MS-plus-self conditions did not affect any of the other psychological measures, such as conscious arousal and feelings. These are in line with previous research showing that mortality salience manipulations do not produce any sign of self-reported affect, anxiety, or arousal changes in participants (Greenberg et al., 1997; Pyszczynski et al., 1999; Arndt et al., 2001). Moreover, our results also suggest that subliminal priming of the concept of death did not lead to an increase in conscious awareness of exerting effort, thus supporting the claim that differences in behavior in the present study are due to largely unconscious mental processes (Aarts et al., 2008).

The current findings advance the understanding of TMT in several ways. According to the self-esteem striving mechanism in TMT, reminders to individuals about their mortality engender responses aimed at increasing self-esteem. Thus, because physical strength is a particularly important domain for men to maintain the meaning and value of the self, it might be expected that mortality salience would directly lead to increased physical strength among men but not women. However, our results did not support this simple hypothesis; male participants in the MS condition did not show an enhancement of physical force. This can be interpreted by a self-escape mechanism buffering against mortality salience, which posits that people low in self-esteem are motivated to escape the self rather than increase cognitions and behaviors associated with bolstering self-esteem when thoughts about death are accessible outside of conscious awareness (Wisman, 2006; Wisman et al., 2015). Because people who lack self-esteem in a certain self-aspect cannot identify more with that construct, they are instead prone to shutting down the self by avoiding self-awareness when reminded of death. Namely, pushing the self away from death can facilitate the suppression of fear of death, defending them from mortality concerns. Considering the fact that the majority of college men have low muscularity esteem (Lynch and Zellner, 1999), male participants in the present study were driven to shut down the self by avoiding self-awareness, and did not engage in behaviors that would bolster their self-esteem to buffer against unconscious mortality salience.

However, a more important finding of the present study is that men’s physical force increased when death-related primes were associated with a self-related stimulus. As mentioned above, people low in self-esteem in a certain aspect, such as their physical strength or physical appearance, are driven to escape the self by avoiding self-awareness of that aspect, as a distal defense against mortality concern. This helps them to assuage unconscious mortality concerns because people can cut off the self from death. However, once death is co-activated and directly associated with the self, people can no longer escape from self-awareness and fear of self-demise, which, in turn, should lead to an attempt to bolster self-esteem. As OSA theory (Duval and Wicklund, 1972) suggests, focusing on the self is one of the important motives of behavior control (Carver and Scheier, 1981, 1998). On the other hand, TMT research suggests that when people low in self-esteem experience mortality concerns, they are prone to shutting down the self by increasing behaviors (e.g., drinking alcohol) aimed at avoiding self-awareness (Wisman, 2006; Wisman et al., 2015). Although those behaviors help them to assuage mortality concerns, it seems that this is just a temporary respite from mortality concerns. Namely, according to the basic premise of TMT, bolstering psychological structures that promote meaning, self-esteem, or immortality eliminates death thoughts (e.g., Arndt et al., 1997b). However, avoiding self-awareness does not provide the essential means that helps one to escape from fear of death. This is because the fear of death may again rise to the surface unless they engage in an action that bolsters the significance of the self. Unlike those low in self-esteem, people with high self-esteem in a certain self-aspect are likely to identify more with that construct and enhance the self as a distal defense to mortality concerns (Landau and Greenberg, 2006). Thus, people high in self-esteem do not have to avoid self-awareness in response to reminders of mortality because they possess the means to cope with mortality concerns by increasing behaviors aimed at bolstering self-esteem. In that sense, the present study suggests the role of the self as a motivational factor underlying TMT processes.

Sport psychologists have searched for ways to unlock the full performance potential of athletes. For example, they have shown that using cognitive and/or somatic techniques, such as asking participants to get themselves emotionally “pumped up” or “charged up,” raises their levels of arousal, resulting in the greatest increases in maximal strength performance (Shelton and Mahoney, 1978; Weinberg et al., 1980; Caudill et al., 1983). However, the present study, based on the perspective of TMT, shows that this effect can occur independently of conscious arousal and awareness. Our findings imply that if individuals are likely to derive self-esteem from their physical strength, a standard mortality salience manipulation without co-activation of the self would be sufficient to increase their physical force. For example, athletes are highly invested in physical performance and are likely to derive self-esteem from their athletic ability (Ellis et al., 2003). In this light, it is evident that reminders of mortality can lead to improved athletic performance in woman athletes as well as in men whose self-esteem is invested in performing. This suggests that trying every kind of sport at the risk of one’s life, not metaphorically, but literally, could lead to better results.

TMT research has demonstrated usefulness in enhancing our understanding of a variety of human behaviors. The current study suggests that TMT may be useful in understanding physical exertion and improving athletic performance. For example, if these findings are replicated not only in handgrip force but also across other domains, future research should consider the pragmatic utility of mortality contemplation as another motivational technique especially for athletes, in addition to psyching-up. However, there are also certainly situations where unlocking men’s physical force would not be a good thing, such as in situations of domestic violence. For instance, people who can derive self-esteem from their physical strength would become more likely to exert greater violence in response to mortality salience. The current findings suggest, in addition, that exerting greater violence in response to mortality reminders may also be mediated by the co-activation of the self even for men with low self-esteem.

### Future Research

There are several limitations which future research should address. First, this study was limited to assessing self-reported, that is, conscious arousal. Therefore, future research should measure unconscious arousal as well. Second, we did not match the neutral and death-related words for frequency and length. Some studies have shown that the length and frequency of words can have a potential confounding effect on behavioral outcomes/cognitive processes (e.g., Hauk and Pulvermüller, 2004). However, our priming procedure to link mortality salience to the self was mainly based on a paradigm used in evaluative conditioning research that has demonstrated robust effects regardless of the length and frequency of words (e.g., Dijksterhuis, 2004; Custers and Aarts, 2005). Nevertheless, we cannot completely rule out the possibility of a confounding effect on physical strength. Future research needs to overcome this limitation by matching the length and frequency of words between the stimulus words. Finally, we acknowledge that relevant individual differences (e.g., body image/satisfaction, body esteem, and drive for muscularity) were not controlled for in this study. However, research has shown that 84% of college men indicated that their current bodies were less muscular than the bodies they would like to possess (Lynch and Zellner, 1999). In this sense, it seems plausible that most participants in our study had the motivation to be muscular based on a cultural ideal, though to differing degrees. Nevertheless, future research that controls these individual differences can shed additional light on the mechanism of enhancing physical strength through mortality salience.

## Author Contributions

NK, EM, and MN designed the study. NK and EM collected and analyzed the data. NK wrote the paper. All authors approved the final version of the manuscript for submission.

## Conflict of Interest Statement

The authors declare that the research was conducted in the absence of any commercial or financial relationships that could be construed as a potential conflict of interest.

## Footnotes

1 We use the term “subliminal” as a technique to present stimuli under the subjective threshold. For example, when a stimulus is presented for a very short duration (e.g., 10–50 ms), participants cannot perceive the stimulus with conscious awareness, resulting in failure to report what they saw. Thus, this stimulus presentation is “subliminal.” Moreover, we use the term “unconscious” for mental processes without conscious awareness. Therefore, when participants were affected by subliminal stimuli, the mental processes are “unconscious.”

2 The 30-ms presentation duration was almost equal to that used in previous studies that involved the subliminal priming of death-related words (Arndt et al., 1997a, 2001, 2002a; Koole and Van den Berg, 2005; Landau et al., 2006).

3 To conduct an independent test on the subliminality of the primed death-related words, twenty additional participants were subjected to the MS and MS-plus-self treatments. Specifically, for each of the 50 trials, participants were asked to indicate whether they saw a word related to death or not. On an average, 53% (SD = 6.3) of the responses were correct, which did not differ significantly from chance, *t* < 1. Furthermore, no differences were observed between the two conditions, *t* < 1. In short, the primed death-related words were subliminal, in that participants could not correctly report the exposed stimuli.
